# A Model of Gravity Vector Measurement Noise for Estimating Accelerometer Bias in Gravity Disturbance Compensation

**DOI:** 10.3390/s18030883

**Published:** 2018-03-16

**Authors:** Junbo Tie, Juliang Cao, Lubing Chang, Shaokun Cai, Meiping Wu, Junxiang Lian

**Affiliations:** 1 College of Mechatronics and Automation, National University of Defense Technology, Changsha 410073, China; tiejunbo11@nudt.edu.cn (J.T.); csk527@163.com (S.C.); wu_meiping@163.com (M.W.); jx_lian@163.com (J.L.); 2 Department of Navigation Engineering, Naval University of Engineering, Wuhan 430000, China; changlubin@163.com

**Keywords:** inertial navigation, gravity disturbance compensation, estimation, accelerometer bias

## Abstract

Compensation of gravity disturbance can improve the precision of inertial navigation, but the effect of compensation will decrease due to the accelerometer bias, and estimation of the accelerometer bias is a crucial issue in gravity disturbance compensation. This paper first investigates the effect of accelerometer bias on gravity disturbance compensation, and the situation in which the accelerometer bias should be estimated is established. The accelerometer bias is estimated from the gravity vector measurement, and a model of measurement noise in gravity vector measurement is built. Based on this model, accelerometer bias is separated from the gravity vector measurement error by the method of least squares. Horizontal gravity disturbances are calculated through EGM2008 spherical harmonic model to build the simulation scene, and the simulation results indicate that precise estimations of the accelerometer bias can be obtained with the proposed method.

## 1. Introduction

An inertial navigation system (INS) is an instrument which can autonomously determine the ship’s attitude, velocity and position based on its self-contained gyroscopes and accelerometers [1,2]. Because of the self-localization feature, INS is not vulnerable to any type of external interference and has wide application in military fields. However, as a dead reckoning approach, the precision of INS will drift with time due to its inherent error sources. The inherent error sources of INS not only include the errors of inertial sensors, but also include the gravity disturbance [3,4]. In recent years, the significant improvement of inertial sensors has left the gravity disturbance as the most important error source in high precision inertial navigation [5,6]. In the future the inertial-sensors-induced position error would be reduced to only a few meters per hour with cold atom interferometry gyroscopes [6], and the gravity disturbance compensation should be considered [7,8,9,10].

**Figure 1:**
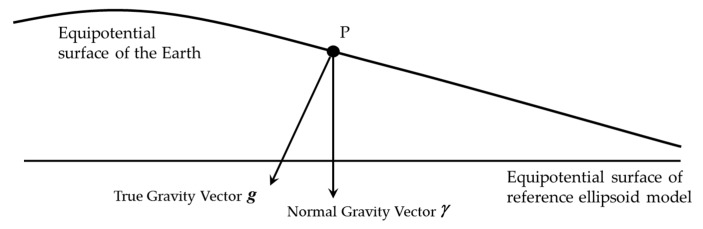
The definition of gravity disturbance vector, gravity disturbance and deflection of vertical, and this figure is adapted from [11].

The definition of gravity disturbance is illustrated in Figure 1 [11]. According to the potential theory, gravity vector is the perpendicular line of equipotential surface of gravity. The Earth’s equipotential surface of gravity is very complex, and the equipotential surface of reference ellipsoid model such as WGS-84, is used to approximate the Earth’s equipotential surface of gravity. As shown in Figure 1, g is the true gravity vector of point P and γ is the normal gravity vector of point P, the gravity disturbance vector is the difference between the true gravity vector and the normal gravity vector [11]. The difference in magnitude is the gravity disturbance and the difference in direction is the deflection of vertical (DOV). Due to DOV, there are some projection components of the true gravity vector in the horizontal plan, which are named horizontal gravity disturbance.

Because of the instability of the INS’s vertical channel, INS generally only provides horizontal velocity, latitude and longitude of the ship, and horizontal gravity disturbance is the main error source of gravity-induced error. The study of compensation of horizontal gravity disturbance can be traced back to the sixties of last century, and works have been done on the analysis of error induced by horizontal gravity disturbance. In 1962, Kayton demonstrated that the practical limit of measurement of inertial acceleration is the knowledge of gravitational field, and any differences between the actual field and the model will cause a navigation error and the use of accelerometers which are more sensitive than 10^−5^ *g* is probably ineffective unless acquiring detailed knowledge of Earth’s gravity field [12]. Based on the work of Kayton, Levine and Gelb quantitatively analyzed the effect of horizontal gravity disturbances on INS, where the horizontal gravity disturbance is modeled as a first-order Gauss-Markov process and the effect on INS is evaluated with the steady-state solution of the covariance matrix differential equation [13]. After that, based on the covariance analysis method proposed by Levine and Gelb, Jordan analyzed the effect of horizontal gravity disturbance with a new model, named self-consistent statistical model. Different from the first-order Gauss-Markov model built by Levine and Gelb, this model is built based on the potential theory, so this model is more consistent with the true covariance of horizontal gravity disturbance [14]. These works indicate that the horizontal gravity disturbance will affect the accuracy of the initial alignment and velocity calculation. In order to compensate the effect on INS, gravity gradiometers were developed to measure the horizontal gravity disturbance along the ship’s trajectory, such as the universal gravity module (UGM) developed by Lockheed Martin Federal Systems [15]. Nowadays, with the release of ultra-high degree global Earth gravitational models, such as the EGM2008, horizontal gravity disturbances can be precisely calculated based on these spherical harmonic models (SHMs), and the effect of horizontal gravity disturbance on INS can be compensated. The compensation method based on SHM is preferable, because only some software updates of INS are needed to obtain the horizontal gravity disturbance, instead of using a costly gradiometer [16,17]. In this paper, calculation of the horizontal gravity disturbance based on SHM is described in detail.

There is a crucial issue in horizontal gravity disturbance compensation, namely the fact that the compensation effect is associated with the accelerometer bias. Because of the tight coupling between accelerometer bias and horizontal gravity disturbance, the method of compensation is ambiguous, especially when considering the initial alignment and INS calculation synthetically. In 1959, the horizontal gravity component from the mission data was used in the MH-311 system for the Army’s AN/USD-5 surveillance. After three years, the derivative of the MH-311 compensates the horizontal gravity disturbance during self-alignment, while removes such compensation in navigation mode. In 1982, an “improved” DOV compensation procedure was used in Mini-GEANS. In particular, the system is firstly aligned to the local gravity vector and after takeoff, the alignment matrix is rotated to the reference ellipsoid. In our early work [18], the compensation is studied in both the initial alignment and navigation calculation. The inertial navigation experiment demonstrates that compensation only in navigation calculation and not in initial alignment is more preferred. Reference [19] reported a military standard ring laser inertial navigation unit named LN-93E, which is an enhanced derivative of its earlier version, LN-93. One of the reasons for the performance improvement of LN-93E is just the DOV compensation at the align location. In [20], the authors analyze why the DOV compensation in initial alignment is not universal. It is pointed out that this is mainly because the correlations between the accelerometer bias and horizontal gravity disturbance. Unfortunately, the corresponding conclusion in [20] is qualitative and the proposed improved compensation procedure is also case-dependent and not universal.

If the accelerometer bias can be accurately estimated, the compensation method can be worked out, and the effect of accelerometer bias on compensation can be eliminated. INS/GNSS (GNSS, global navigation satellite system) integrated is the usual method to estimate the accelerometer bias [21]. However, the precision of this method is limited by the poor observability of accelerometer bias [22,23], and the estimation is the combination of accelerometer bias and horizontal gravity disturbance. Although a statistical model of the horizontal gravity disturbance can be used in the filter to separate the accelerometer bias from the horizontal gravity disturbance, it’s hard to build a universal statistical model of horizontal gravity disturbance [24]. 

In some cases, the use of GNSS is not possible and the positioning can only depend on INS. To improve the accuracy of INS in long-endurance navigation, the compensation of gravity disturbance is very necessary. However, the effect of compensation will decrease due to the accelerometer bias. One possible solution to this problem is estimating the accelerometer bias with GNSS before the long-endurance inertial navigation. In this paper, we try to do the estimation in the method of gravity vector measurement [25,26], we attempt to separate the accelerometer bias from the measurement error of gravity vector. The model of measurement noise is built to implement the estimation. The contents are organized as follows: the horizontal gravity disturbance calculation using SHM is described in Section 2. In Section 3, the reason why accelerometer bias influences compensation is analyzed. In Section 4, our proposed method of estimating accelerometer bias is introduced in detail and simulation results are provided in Section 5. Finally, conclusions are drawn in Section 6.

## 2. Horizontal Gravity Disturbance and Spherical Harmonic Model

### 2.1. Definition of Horizontal Gravity Disturbance

**Figure 2:**
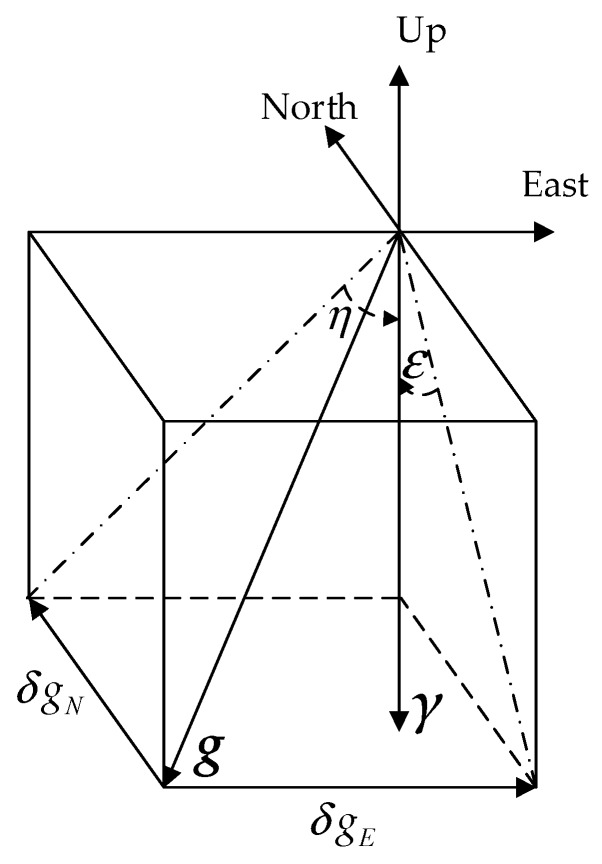
The definition of deflection of vertical and horizontal gravity disturbance.

DOV has two components as shown in Figure 2, where ***g*** is the true gravity vector, and ***γ*** is the normal gravity vector obtained from the reference ellipsoid model such as WGS-84. The DOV, which is a vector quantity, is usually decomposed into two mutually perpendicular components: a north-south or meridional component *ξ*, which is reckoned positive northward, and an east-west or prime vertical component *η*, which is reckoned positive eastward [27]. In other words, the deflection components are positive if the direction of the gravity vector points further south and further west than the corresponding ellipsoidal normal [28], or the level surface is rising to the south or west, respectively, with respect to the ellipsoid [29].

The north component of horizontal gravity disturbance $\Delta g_{N}^{n}$ is associated with *ξ*, and the east component of horizontal gravity disturbance $\Delta g_{E}^{n}$ is associated with *η*. As shown in Figure 2, Equation (1) describes the connections between DOV and horizontal gravity disturbance where *γ* is the norm of the normal gravity vector ***γ***:

(1) $$\begin{array}{cc} \tan\xi \approx \xi =-\frac{\Delta g_{N}^{n}}{\gamma } & \tan\eta \approx \eta =-\frac{\Delta g_{E}^{n}}{\gamma } \end{array}$$

### 2.2. Calculation of Horizontal Gravity Disturbance Based on SHM

According to the potential theory [11], DOV is the partial derivative of the disturbed gravitational potential *T*, and *r* is the distance from the center of reference ellipsoid to the calculated point:

(2) $$\begin{array}{cc} \xi =-\frac{1}{\gamma \cdot r}\frac{\partial T}{\partial L} & \eta =-\frac{1}{\gamma \cdot r\cdot \cos L}\frac{\partial T}{\partial \lambda } \end{array}$$

Substituting Equation (2) into Equation (1), the connections between the disturbed gravitational potential and the horizontal gravity disturbance can be built, and *L* is the geocentric latitude of calculated point and *λ* is longitude of the calculated point:

(3) $$\begin{array}{cc} \Delta g_{N}^{n}=\frac{1}{r}\frac{\partial T}{\partial L} & \Delta g_{E}^{n}=\frac{1}{r\cdot \cos L}\frac{\partial T}{\partial \lambda } \end{array}$$

Usually the geocentric colatitude *ϑ* is used in the SHM calculation:

(4) $$\vartheta =\frac{\pi }{2}-L$$

Then the horizontal gravity disturbances can be obtained as:

(5) $$\Delta g_{N}^{n}=-\frac{1}{r}\frac{\partial T}{\partial \vartheta }$$

(6) $$\Delta g_{E}^{n}=\frac{1}{r\cdot \sin\vartheta }\frac{\partial T}{\partial \lambda }$$

The disturbed gravitational potential *T* is the solution of Laplace equation in the ellipsoidal coordinate frame, which can be represented as follows [11]:

(7) $$T=\frac{GM}{r}{\sum_{n=2}^{nmax}\sum_{m=0}^{n}\left(\frac{a}{r}\right)}^{n}\left({\bar{C}}_{nm}^{*}\cos m\lambda +{\bar{S}}_{nm}\sin m\lambda \right){\bar{P}}_{nm}\left(\cos\vartheta \right)$$

where *G* is the gravitational constant, *M* is the mass of the Earth, *a* is the major semi-axis length of the reference ellipsoid, *n* and *m* are called the degree and order of the SHM, ${\bar{C}}_{nm}^{*}$ and ${\bar{S}}_{nm}$ are the coefficients of the SHM, ${\bar{P}}_{nm}\left(\cos\vartheta \right)$ is the fully normalized Legendre functions of degree *n* and order *m*. The partial derivatives of the disturbed gravitation potential are Equations (8) and (9) [11]:

(8) $$\frac{\partial T}{\partial \vartheta }=\frac{GM}{r}\sum_{n=2}^{nmax}\sum_{m=0}^{n}{\left(\frac{a}{r}\right)}^{n}\left({\bar{C}}_{nm}^{*}\cdot \cos m\lambda +{\bar{S}}_{nm}\cdot \sin m\lambda \right)\frac{d{\bar{P}}_{nm}\left(\cos\vartheta \right)}{d\vartheta }$$

(9) $$\frac{\partial T}{\partial \lambda }=\frac{GM}{r}\sum_{n=2}^{nmax}\sum_{m=0}^{n}{\left(\frac{a}{r}\right)}^{n}\left[m\left(-{\bar{C}}_{nm}^{*}\cdot \sin m\lambda +{\bar{S}}_{nm}\cdot \cos m\lambda \right)\right]{\bar{P}}_{nm}\left(\cos\vartheta \right)$$

Substituting Equations (8) and (9) into Equations (5) and (6), the formulas of calculating horizontal gravity disturbance are obtained:

(10) $$\Delta g_{N}^{n}=-\frac{GM}{r^{2}}\sum_{n=2}^{nmax}\sum_{m=0}^{n}{\left(\frac{a}{r}\right)}^{n}\left({\bar{C}}_{nm}^{*}\cdot \cos m\lambda +{\bar{S}}_{nm}\cdot \sin m\lambda \right)\frac{d{\bar{P}}_{nm}\left(\cos\vartheta \right)}{d\vartheta }$$

(11) $$\Delta g_{E}^{n}=\frac{GM}{\sin\vartheta \cdot r^{2}}\sum_{n=2}^{nmax}\sum_{m=0}^{n}{\left(\frac{a}{r}\right)}^{n}\left[m\left(-{\bar{C}}_{nm}^{*}\cdot \sin m\lambda +{\bar{S}}_{nm}\cdot \cos m\lambda \right)\right]{\bar{P}}_{nm}\left(\cos\vartheta \right)$$

## 3. The Effect of Accelerometer Bias on Horizontal Gravity Disturbance Compensation

### 3.1. Reference Coordinate Frames

#### 3.1.1. Earth-Centered-Earth-Fixed Frame *e*

**Figure 3:**
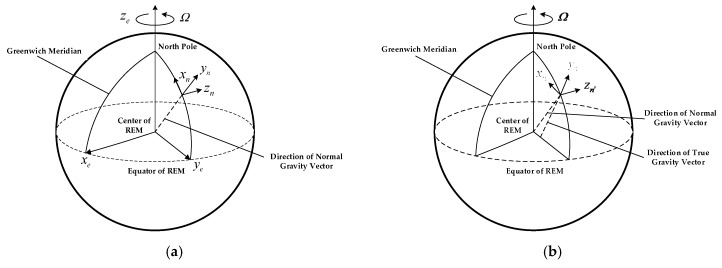
The definition of navigation coordinate frame, (**a**) is the diagram of *n* and (**b**) is the diagram of *n′*.

The origin of this coordinate frame is at center of the Earth, whose *z*-axis points in the direction of the North pole, *x*-axis points towards the Greenwich Meridian, and *y*-axis completes the right-handed orthogonal frame. This frame rotates with the Earth with the rate $\omega_{ie}^{e}={\left[0\;0\;\Omega \right]}^{T}$, Ω is the Earth’s rotation angular velocity, as shown in Figure 3a.

#### 3.1.2. Navigation Coordinate Frame with North-Up-East Definition *n*

This frame is a local geodetic north-oriented, local-level coordinate frame, the origin of this frame is at the position of the ship, and its *x_n_−y_n_−z_n_* axes respectively point towards North-Up-East, as shown in Figure 3a. It should be noted that *z_n_* is collinear with the normal gravity vector which points towards the center of the reference ellipsoid.

#### 3.1.3. Body Coordinate Frame with Forward-Upward-Right *b*

This frame is defined based on the input axes of inertial sensors. Its axes respectively point towards Right-Forward-Upward of the ship.

#### 3.1.4. Navigation Coordinate Frame with True Vertical *n’*

This frame is similar to n coordinate frame. Based on the true gravity vector, whose axes are denoted by { *x_n′_,y_n′_,z_n′_*} and *x_n′_* also points towards north, but *z_n′_* is collinear with the true gravity vector and *y_n′_* can be determined based on the right-hand rule, as shown in Figure 3b.

Direction cosine matrix (DCM) is used to express a rotation in three dimensions as a mathematical transformation. The DCM between navigation frame and body frame is a function of Euler angles:

(12) $$r^{n}=C_{b}^{n}r^{b}$$

where $r^{n}$ is the projection of arbitrary vector r in n coordinate frame, $r^{b}$ is the projection of vector r in b coordinate frame.

### 3.2. Mathmatical Formulation of INS

**Figure 4:**
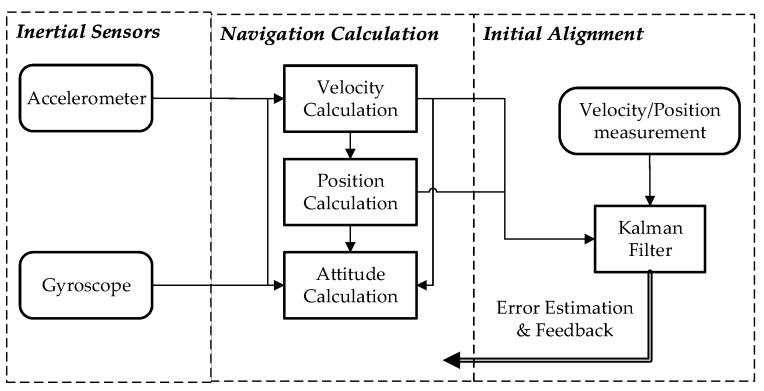
The schematic of inertial navigation.

Inertial navigation is an integration algorithm based on Newton’s second law. It can be decomposed into two successive steps as shown in Figure 4. Step I is the initial alignment in which the initial values of the integration algorithm are obtained, including initial attitudes, initial velocities, and initial positions. Step II is the integration calculation named navigation calculation, including attitude calculation, velocity calculation and position calculation. In fact, the implementation of step I contains step II. Navigation calculation is first executed in step I, then Kalman Filter (KF) recursion is performed following the one-step navigation calculation. After the KF measurement update, the estimated state can be fed back to fix the corresponding navigation calculation errors. Finally, the precise initial values will be obtained in the KF recursion.

Mechanization is very important for inertial navigation and the analysis in this paper. The traditional INS strapdown mechanization is chosen in this paper for two reasons. Firstly, as introduced in Section 1, the background of this paper is compensation of horizontal gravity disturbance for high precision INS. Secondly, the accelerometer bias is estimated from the strapdown gravity vector measurement, and in the field of strapdown gravity vector measurement, the traditional INS strapdown mechanization is appropriate [30,31,32].

The navigation calculation is performed in the navigation frame and all the vectors should be transformed into this frame before they can be used. Navigation coordinate frame with north-up-east definition is usually chosen as the coordinate frame in which the navigation calculation is implemented.

The attitude kinematical equation with DCM parameterization is given by [2]:

(13) $${\dot{C}}_{b}^{n}=C_{b}^{n}\left[\omega_{nb}^{b}\times \right]$$

where $\omega_{nb}^{b}$ is the body angular rate with respect to the navigation frame *n* and is given by [2]:

(14) $$\omega_{nb}^{b}=\omega_{ib}^{b}-C_{n}^{b}\left(\omega_{ie}^{n}+\omega_{en}^{n}\right)$$

$\omega_{ib}^{b}$ is the body angular rate with respect to inertial frame and is measured by the gyroscopes. $\omega_{ie}^{n}$ is the earth rotational rate and is given by [2]:

(15) $$\omega_{ie}^{n}={\left[\begin{array}{ccc} \Omega \cos L & \Omega sinL & 0 \end{array}\right]}^{T}$$

$\omega_{ie}^{n}$ is the angular rate of navigation frame with respect to the Earth frame, which is caused by the linear motion of the ship on the ellipsoidal surface. The formulation of $\omega_{ie}^{n}$ is given by [2]:

(16) $$\omega_{en}^{n}={\left[\begin{array}{ccc} \frac{v_{E}^{n}}{R_{N}+h} & \frac{v_{E}^{n}}{R_{N}+h}\tan L & -\frac{v_{N}^{n}}{R_{M}+h} \end{array}\right]}^{T}$$

$R_{M}$ and $R_{N}$ are the meridian and transverse radius of the ellipsoid curvature, respectively. $v_{E}^{n}$ and $v_{N}^{n}$ are the east and north components of the velocity, respectively. *h* is the height of the ship relative to the reference ellipsoid, and it should be noted that this paper focuses on the ship mounted INS and the height can be set to zero.

The velocity kinematical equation in navigation frame is given by [2]:

(17) $${\dot{v}}_{e}^{n}=C_{b}^{n}f^{b}-(2\omega_{ie}^{n}+\omega_{en}^{n})\times v_{e}^{n}+g^{n}$$

where $v_{e}^{n}$ is the velocity relative to the Earth, $f^{b}$ is the specific force measured by the accelerometers, and it’s emphasized here that $g^{n}$ is the true gravity vector at the position of ship. $\Delta g^{n}$ is the horizontal gravity disturbance described in Section 2, and $\gamma^{n}$ is the normal gravity vector obtained from reference ellipsoid model:

(18) $$g^{n}=\left[\begin{array}{c} \Delta g_{N}^{n}\\ -\gamma \\ \Delta g_{E}^{n} \end{array}\right]=\left[\begin{array}{c} \Delta g_{N}^{n}\\ 0\\ \Delta g_{E}^{n} \end{array}\right]+\left[\begin{array}{c} 0\\ -\gamma \\ 0 \end{array}\right]=\Delta g^{n}+\gamma^{n}$$

The definition of horizontal gravity disturbance compensation can be defined here, that is horizontal gravity disturbance compensation means that the gravity vector used in initial alignment and navigation calculation is $g^{n}$ rather than $\gamma^{n}$. 

The position kinematical equation is given here and the kinematical equation of height is not considered here for ship mount INS [2]:

(19) $$\begin{array}{l} \dot{L}=\frac{1}{R_{M}+h}v_{N}^{n}\\ \dot{\lambda }=\frac{\sec L}{R_{N}+h}v_{E}^{n} \end{array}$$

### 3.3. Effect of Acceleromter Bias on Compensation

Attitude error equation and velocity error equation are the foundations of KF recursion in initial alignment and are also the key point of the analysis. The attitude error equation is given in [2]:

(20) $$\delta \varphi ={\left[\begin{array}{ccc} \delta \alpha & \delta \phi & \delta \beta \end{array}\right]}^{T}$$

(21) $$\delta \dot{\varphi }=-\left(\omega_{ie}^{n}+\omega_{en}^{n}\right)\times \delta \varphi +\delta \omega_{in}^{n}-C_{b}^{n}\delta \omega_{ib}^{b}$$

where δφ is the attitude error vector, δα is the roll error, δϕ is the yaw error, δβ is the pitch error and $\delta \omega_{ib}^{b}$ is the gyroscope noise which can be usually regarded as white noise.

The velocity error equation is derived as follows. The true velocity kinematic equation is given in [2], as Equation (22):

(22) $${\dot{v}}_{e}^{n}=C_{b}^{n}f^{b}-(2\omega_{ie}^{n}+\omega_{en}^{n})\times v_{e}^{n}+\gamma^{n}+\Delta g^{n}$$

When the navigation calculation is implemented without the gravity disturbance compensation and no accelerometer bias exists, the practical velocity kinematic equation in navigation calculation is Equation (23):

(23) $${\dot{\tilde{v}}}_{e}^{n}={\tilde{C}}_{b}^{n}f^{b}-(2{\tilde{\omega }}_{ie}^{n}+{\tilde{\omega }}_{en}^{n})\times {\tilde{v}}_{e}^{n}+\gamma^{n}+\delta f^{n}$$

where ${\tilde{v}}_{e}^{n}$ is the calculated velocity-containing error. ${\tilde{\omega }}_{ie}^{n}$ and ${\tilde{\omega }}_{en}^{n}$ are angular velocities containing error, $\gamma^{n}$ is the normal gravity vector, and $\delta f^{n}$ is the white noise in accelerometer output. ${\tilde{C}}_{b}^{n}$ is the DCM containing error and is defined as follows:

(24) $${\tilde{C}}_{b}^{n}=\left(I_{3\times 3}-\left[\delta \varphi \times \right]\right)C_{b}^{n}$$

(25) $$\left[\delta \varphi \times \right]=\left[\begin{array}{ccc} 0 & -\delta \beta & \delta \phi \\ \delta \beta & 0 & -\delta \alpha \\ -\delta \phi & \delta \alpha & 0 \end{array}\right]$$

The velocity error equation without accelerometer bias is derived by subtracting Equation (22) from Equation (23):

(26) $$\delta v_{e}^{n}={\tilde{v}}_{e}^{n}-v_{e}^{n}={\left[\begin{array}{ccc} \delta v_{N}^{n} & \delta v_{U}^{n} & \delta v_{E}^{n} \end{array}\right]}^{T}$$

(27) $$\left[f^{n}\times \right]=\left[\begin{array}{ccc} 0 & -f_{E}^{n} & f_{U}^{n}\\ f_{E}^{n} & 0 & -f_{N}^{n}\\ -f_{U}^{n} & f_{N}^{n} & 0 \end{array}\right]$$

(28) $$\delta {\dot{v}}_{e}^{n}=\left[f^{n}\times \right]\delta \varphi +\delta f^{n}-\Delta g^{n}$$

where $\delta v_{e}^{n}$ is the velocity error vector, $\delta v_{N}^{n}$, $\delta v_{U}^{n}$ and $\delta v_{E}^{n}$ are the north, upward and east component of velocity error vector. $f^{n}$ is the specific force measured at the initial point and projected in the *n* frame. $f_{N}^{n}$, $f_{U}^{n}$ and $f_{E}^{n}$ are the components of ***f****^n^* in the *n* frame. 

Initial alignment is usually implemented when the INS is at static or mooring, ***f****^n^* can be regarded as the opposite of the true gravity vector:

(29) $$f^{n}=-g^{n}={\left[\begin{array}{ccc} -\Delta g_{N}^{n} & \gamma & -\Delta g_{E}^{n} \end{array}\right]}^{T}$$

The Kalman filter state is updated with a new observation, velocity error is usually used as observation in static initial alignment. Because the initial alignment is implemented when the ship is at static or moored, the true value of velocity is approximately equal to zero, then the non-zero velocity output of INS is the velocity error. KF recursion of initial alignment will converge when the velocity error reaches zero, and the attitude estimation errors can be obtained by setting Equation (28) equal to zero:

(30) $$\delta \alpha \approx \frac{\Delta g_{E}^{n}}{\gamma }=\frac{-\eta \cdot \gamma }{\gamma }=-\eta$$

(31) $$\delta \beta \approx \frac{-\Delta g_{N}^{n}}{\gamma }=\frac{\gamma \cdot \xi }{\gamma }=\xi$$

From Equations (30) and (31), it can be seen that horizontal gravity disturbance will give rise to the attitude errors in the initial alignment. Compensation for horizontal gravity disturbance is necessary in initial alignment. 

However, if some accelerometer bias exists, the coupling between accelerometer bias and horizontal gravity disturbance may decrease the compensation effect as analyzed below. When navigation calculation is implemented without gravity disturbance compensation and accelerometer bias exists, the velocity kinematic equation is Equation (32)

(32) $${\dot{\tilde{v}}}_{e}^{n}={\tilde{C}}_{b}^{n}f^{b}-(2{\tilde{\omega }}_{ie}^{n}+{\tilde{\omega }}_{en}^{n})\times {\tilde{v}}_{e}^{n}+\gamma^{n}+\delta f^{n}+b_{a}^{n}$$

The new velocity error equation is obtained by subtracting Equation (22) from Equation (32):

(33) $$b_{a}^{n}={\left[\begin{array}{ccc} b_{a,N}^{n} & b_{a,U}^{n} & b_{a,E}^{n} \end{array}\right]}^{T}$$

(34) $$\delta {\dot{v}}_{e}^{n}=\left[f^{n}\times \right]\delta \varphi +\delta f^{n}-\Delta g^{n}+b_{a}^{n}$$

where $b_{a}^{n}$ is the accelerometer bias and $b_{a,N}^{n}$, $b_{a,U}^{n}$ and $b_{a,E}^{n}$ are the north, upward and east component of the accelerometer bias. The estimation errors of attitude are obtained as Equations (35) and (36) which clearly describe the connection among attitude error, horizontal gravity disturbance and accelerometer bias:

(35) $$\delta \alpha \approx \frac{\Delta g_{E}^{n}+b_{a,E}^{n}}{\gamma }=\frac{-\eta \cdot \gamma }{\gamma }+\frac{b_{a,E}^{n}}{\gamma }=-\eta +\frac{b_{a,E}^{n}}{\gamma }$$

(36) $$\delta \beta \approx \frac{-\Delta g_{N}^{n}+b_{a,N}^{n}}{\gamma }=\frac{\gamma \cdot \xi }{\gamma }+\frac{b_{a,N}^{n}}{\gamma }=\xi +\frac{b_{a,N}^{n}}{\gamma }$$

In the case of the accelerometer bias being much larger than the horizontal gravity disturbance, whether the horizontal gravity disturbance being compensated will not obviously improve the precision of initial alignment, because the accelerometer bias is the main error source. The accelerometer bias can be estimated through INS/GNSS integrated Kalman filter [21].

In the case of the accelerometer bias being much smaller than the horizontal gravity disturbance, the compensation of horizontal gravity disturbance which is the dominant error source will reliably improve the accuracy of initial alignment, and there is no need to estimate accelerometer bias in practice.

In the case of accelerometer bias being at the same order with horizontal gravity disturbance, this is the hardest situation to handle. Taking Equation (35) for example, if the sign and magnitude of east accelerometer bias are the same as those of east gravity disturbance, the effect of horizontal gravity disturbance on initial alignment is counteracted with the accelerometer bias. If the gravity disturbance is still compensated in initial alignment, then a new attitude error will arise due to the compensation. Therefore, estimation of accelerometer bias is necessary in this situation, otherwise whether do the gravity disturbance compensation in initial alignment will be ambiguous as mentioned in the introduction.

The above analysis also shows the effect of horizontal gravity disturbance on navigation calculation. As in Equation (32), the accelerometer bias is coupled with the horizontal gravity disturbance in the velocity calculation. When the horizontal gravity disturbance is compensated, the practical kinematic equation is Equation (37):

(37) $${\dot{\tilde{v}}}_{e}^{n}={\tilde{C}}_{b}^{n}f^{b}-(2{\tilde{\omega }}_{ie}^{n}+{\tilde{\omega }}_{en}^{n})\times {\tilde{v}}_{e}^{n}+\gamma^{n}+\Delta g^{n}+\delta f^{n}+b_{a}{}^{n}$$

Comparing Equation (37) with Equation (22), it can be found that the effect of compensation is counteracted with the accelerometer bias. In the limit situation of the accelerometer bias being the opposite of horizontal gravity disturbance, the compensation will be inefficient. 

## 4. Model of Gravity Vector Measurement Noise

From the analysis in Section 3, when the accelerometer bias is at the same order with horizontal gravity disturbance, accurate estimation of accelerometer bias is the crucial issue in gravity disturbance compensation. Usually the accelerometer bias can be estimated using INS/GNSS integrated Kalman filter in which the accelerometer bias is modeled as a constant. The disadvantage to this approach is that the estimation is a combination of the accelerometer bias and horizontal gravity disturbance. Maybe we can try to do the estimation from another angle. Inertial sensors not only can be used to navigate but also can be used to measure the gravity vector. If the gravity information is the input, position information is obtained. On the contrary, gravity vector can be measured as position and velocity information being the input, that’s the principle of the strapdown gravimeter [25].

Horizontal gravity disturbance can be measured by subtracting the normal gravity vector from the measured value of gravity vector as Equation (38):

(38) $$\Delta g^{n}+w={\dot{v}}_{e}^{n}-C_{b}^{n}f^{b}+\left(2\omega_{ie}^{n}+\omega_{en}^{n}\right)\times v_{e}^{n}-\gamma^{n}$$

Equation (38) is the transformation of Equation (22). In gravity vector measurement, some terms of Equation (38) is calculated based on GNSS information, and some are provided by inertial sensors. ${\dot{v}}_{e}^{n}$ is the difference of velocity provided by GNSS, $\omega_{ie}^{n}$ and $\omega_{en}^{n}$ can be calculated by substituting the velocity and position provided by GNSS into Equation (15) and Equation (16). $C_{b}^{n}$ is calculated with the output of gyroscopes and Equation (13). $f^{b}$ is the output of the accelerometers. 

The precise measurement of specific force is crucial for gravity vector measurement and INS is aided with GNSS to improve attitude accuracy, thus high precise measurement of specific force can be obtained. As described in [33], there are four main data fusion schemes for INS/GNSS. In the traditional strapdown gravity vector measurement, feedback correction is the main data fusion scheme of INS/GNSS which is also the data fusion scheme adopted in this paper. And a new and compact data fusion scheme proposed in [33] is a novel and worthwhile approach in the advancement of strapdown gravity vector measurement.

In addition, it should be noted that w is the measurement noise of gravity vector, which is a composite term due to some error sources except accelerometer bias, including noise of inertial sensors and error of GNSS. The measured value of horizontal gravity disturbance can be expressed as follows:

(39) $$\begin{array}{cc} \Delta g_{N,E}^{n}={\left[\begin{array}{cc} \Delta g_{N}^{n} & \Delta g_{E}^{n} \end{array}\right]}^{T} & w={\left[\begin{array}{cc} w_{N} & w_{E} \end{array}\right]}^{T} \end{array}$$

(40) $$\Delta {\tilde{g}}_{N,E}^{n}=\Delta g_{N,E}^{n}+w$$

where $\Delta g_{N,E}^{n}$ is the true value of horizontal gravity disturbance, $\Delta {\tilde{g}}_{N,E}^{n}$ is the measured value containing error, w is the measurement noise. When the accelerometer bias exits, the accelerometer bias is added to the measurement equation and the measured value also changes:

(41) $$\Delta g^{n}+w+\Delta f^{n}={\dot{v}}_{e}^{n}-C_{b}^{n}f^{b}+\left(2\omega_{ie}^{n}+\omega_{en}^{n}\right)\times v_{e}^{n}-\gamma^{n}$$

(42) $$\Delta {\tilde{g}}_{N,E}^{n}=\Delta g_{N,E}^{n}+w+{\left[\begin{array}{cc} b_{a,N}^{n} & b_{a,E}^{n} \end{array}\right]}^{T}$$

The measurement error of horizontal gravity disturbance can be obtained by subtracting the measured value from the true value:

(43) $$\delta g_{N,E}^{n}=\left[\begin{array}{c} \delta g_{N}^{n}\\ \delta g_{E}^{n} \end{array}\right]=\left[\begin{array}{c} b_{a,N}^{n}\\ b_{a,E}^{n} \end{array}\right]+\left[\begin{array}{c} w_{N}\\ w_{E} \end{array}\right]$$

where $\delta g_{N}^{n}$ and $\delta g_{E}^{n}$ are the north and east component of measurement error of horizontal gravity disturbance. Because the background of this paper is using the calculated horizontal gravity disturbance based on SHM to compensate INS, the horizontal gravity disturbance from SHM is regarded as the true value, the measurement error can be calculated by subtracting the true value from the measured value.

In fact, we can have estimations of accelerometer biases by averaging the measurement error as follows:

(44) $$\begin{array}{cc} {\hat{b}}_{a,N}^{n}=\frac{1}{N}\sum_{i=1}^{N}\left[\delta g_{N}^{n}\left(i\right)\right] & {\hat{b}}_{a,E}^{n}=\frac{1}{N}\sum_{i=1}^{N}\left[\delta g_{E}^{n}\left(i\right)\right] \end{array}$$

${\hat{b}}_{a,N}^{n}$ and ${\hat{b}}_{a,E}^{n}$ will be precise estimations if and only if the measurement noise is zero-mean, but it is a too rigorous condition for practice. Based on the Equation (44), when the mean value of measurement noise isn’t zero, is it possible to have a precise estimation of the accelerometer bias? The conjecture is that accelerometer bias is the inherent error of accelerometer which is not related to the Earth’s gravity field, but the contribution of measurement noise to measurement error may be associated with the gravity field. 

### 4.1. The Measurement Noise of Horizontal Gravity Disturbance

In this subsection, the model of measurement noise will be derived. The true gravity vector projected in n coordinate frame is Equation (18), while the true gravity vector projected in $n^{\prime }$ coordinate frame is Equation (45):

(45) $$g^{n^{\prime }}={\left[\begin{array}{ccc} 0 & -\gamma & 0 \end{array}\right]}^{T}$$

**Figure 5:**
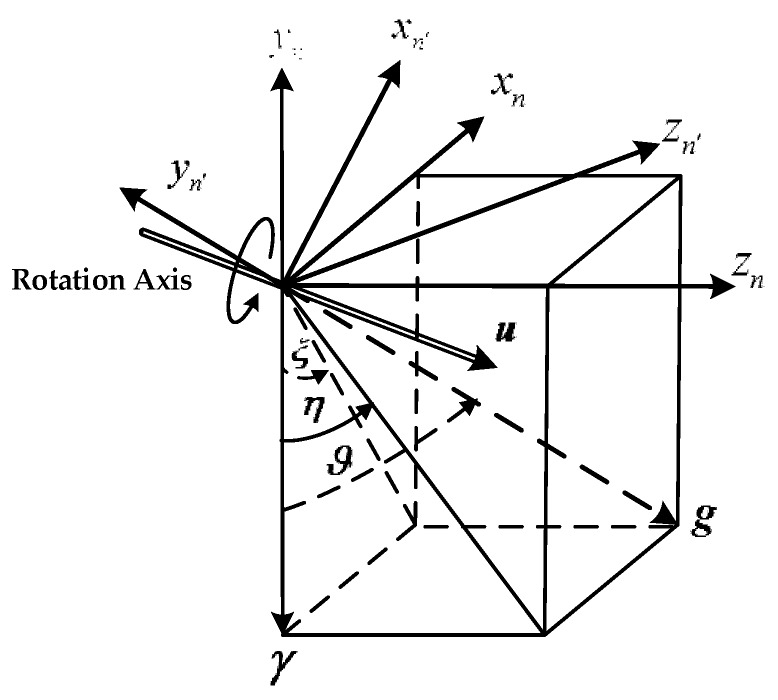
The geometric relationship between the two navigation coordinate frames.

It should be noted that the *y*-axis of $n^{\prime }$ coordinate frame is collinear with the true gravity vector, the horizontal components of $g^{n^{\prime }}$ are zeroes. The DCM between n coordinate frame and $n^{\prime }$ coordinate frame can be determined based on the geometric relationship between the two navigation coordinate frames, as shown in Figure 5. 

The coordinate frame *n′* can be obtained through rotating the coordinate frame *n* by ϑ along the rotational axis ***u***. It is obvious that the rotational axis and rotational angle are associated with the DOV components and can be determined based on some constraints. The rotation axis $u^{n}={\left[\begin{array}{ccc} u_{x} & u_{y} & u_{z} \end{array}\right]}^{T}$ satisfies four constraints:

(1) In the plane $x_{n}-z_{n}$;
(2) Pass through the origin of the two coordinate frames;
(3) Be orthogonal to the plane $y_{n}-y_{n^{\prime }}$;
(4) u is unit vector;

Based on constraint 1:

(46) $$u_{y}=0$$

Based on constraint 2, *k* is a constant to be determined:

(47) $$u_{x}=k\cdot u_{z}$$

Based on constraint 3:

(48) $$u^{n}\cdot g^{n}={\left[\begin{array}{ccc} u_{x} & u_{y} & u_{z} \end{array}\right]}^{T}\cdot {\left[\begin{array}{ccc} \varepsilon \cdot \gamma & -\gamma & \eta \cdot \gamma \end{array}\right]}^{T}=0$$

Based on constraint 4:

(49) $$\sqrt{u_{x}^{2}+u_{y}^{2}+u_{z}^{2}}=1$$

According to Equations (46)–(49), the rotation axis ***u*** can be determined:

(50) $$\begin{array}{ccc} u_{x}=\left(-\eta \right)/\sqrt{\xi^{2}+\eta^{2}} & u_{y}=0 & u_{z}=\xi /\sqrt{\xi^{2}+\eta^{2}} \end{array}$$

(51) $$u=\left[\begin{array}{ccc} u_{x} & 0 & u_{z} \end{array}\right]={\left[\begin{array}{ccc} -\eta /\sqrt{\xi^{2}+\eta^{2}} & 0 & \xi /\sqrt{\xi^{2}+\eta^{2}} \end{array}\right]}^{T}$$

ϑ is the angle between $\gamma^{n}$ and $g^{n}$, and can be calculated based on vector product:

(52) $$\begin{array}{ccc} \gamma^{n}={\left[\begin{array}{ccc} 0 & -\gamma & 0 \end{array}\right]}^{T} & g^{n}=\left[\begin{array}{ccc} \delta g_{N} & -\gamma & \delta g_{E} \end{array}\right] & \cos\vartheta =\left(\bar{g}\cdot g\right)/\left(\left\|\bar{g}\right\|\cdot \left\|g\right\|\right) \end{array}$$

(53) $$\vartheta =\arccos\left(1/\sqrt{1+\xi^{2}+\eta^{2}}\right)$$

Based on the rotation axis and the rotation angle, the corresponding quaternion and DCM can be obtained [2]:

(54) $$Q=\left[\begin{array}{cccc} q_{0} & q_{1} & q_{2} & q_{3} \end{array}\right]=\left[\begin{array}{cccc} \cos\left(\vartheta /2\right) & u_{x}\cdot \cos\left(\vartheta /2\right) & u_{y}\cdot \cos\left(\vartheta /2\right) & u_{z}\cdot \cos\left(\vartheta /2\right) \end{array}\right]$$

(55) $$C_{n^{\prime }}^{n}=\left[\begin{array}{ccc} q_{0}^{2}+q_{1}^{2}-q_{2}^{2}-q_{3}^{2} & 2\left(q_{1}\cdot q_{2}-q_{0}\cdot q_{3}\right) & 2\left(q_{1}\cdot q_{3}+q_{0}\cdot q_{2}\right)\\ 2\left(q_{1}\cdot q_{2}+q_{0}\cdot q_{3}\right) & q_{0}^{2}-q_{1}^{2}+q_{2}^{2}-q_{3}^{2} & 2\left(q_{2}\cdot q_{3}-q_{0}\cdot q_{1}\right)\\ 2\left(q_{1}\cdot q_{3}-q_{0}\cdot q_{2}\right) & 2\left(q_{2}\cdot q_{3}+q_{0}\cdot q_{1}\right) & q_{0}^{2}-q_{1}^{2}-q_{2}^{2}+q_{3}^{2} \end{array}\right]$$

Equations (54) and (55) can be simplified due to *u_y_* being zero:

(56) $$Q=\left[\begin{array}{cccc} \cos\left(\vartheta /2\right) & u_{x}\cdot \cos\left(\vartheta /2\right) & 0 & u_{z}\cdot \cos\left(\vartheta /2\right) \end{array}\right]$$

(57) $$C_{n^{\prime }}^{n}=\left[\begin{array}{ccc} q_{0}^{2}+q_{1}^{2}-q_{3}^{2} & -2\left(q_{0}\cdot q_{3}\right) & 2\left(q_{1}\cdot q_{3}\right)\\ 2\left(q_{0}\cdot q_{3}\right) & q_{0}^{2}-q_{1}^{2}-q_{3}^{2} & 2\left(-q_{0}\cdot q_{1}\right)\\ 2\left(q_{1}\cdot q_{3}\right) & 2\left(q_{0}\cdot q_{1}\right) & q_{0}^{2}-q_{1}^{2}+q_{3}^{2} \end{array}\right]$$

Based on the Equations (50)–(57), it can be seen that $C_{n^{\prime }}^{n}$ is a function of the horizontal gravity disturbance:

(58) $$C_{n^{\prime }}^{n}=F\left(\Delta g_{N}^{n},\Delta g_{E}^{n}\right)$$

and Equation (18) can be approximately rearranged as:

(59) $$C_{n^{\prime }}^{n}g^{n^{\prime }}=C_{n^{\prime }}^{n}\left[\begin{array}{c} 0\\ -\gamma \\ 0 \end{array}\right]=g^{n}=\left[\begin{array}{c} \Delta g_{N}^{n}\\ -\gamma \\ \Delta g_{E}^{n} \end{array}\right]$$

Now, when only considering the measurement noise, the DCM based on the measured values is supposed to has the following form:

(60) $$F\left(\Delta g_{N}^{n}+w_{N},\Delta g_{E}^{n}+w_{E}\right)={\tilde{C}}_{n^{\prime }}^{n}=\left(C_{n^{\prime }}^{n}+\delta C_{n^{\prime }}^{n}\right)$$

Substituting Equation (60) into Equation (59):

(61) $$\left[\begin{array}{c} \Delta g_{N}^{n}+w_{N}\\ -\gamma \\ \Delta g_{E}^{n}+w_{E} \end{array}\right]\approx {\tilde{C}}_{n^{\prime }}^{n}\left[\begin{array}{c} 0\\ -\gamma \\ 0 \end{array}\right]=\left(C_{n^{\prime }}^{n}+\delta C_{n^{\prime }}^{n}\right)\left[\begin{array}{c} 0\\ -\gamma \\ 0 \end{array}\right]=C_{n^{\prime }}^{n}\left[\begin{array}{c} 0\\ -\gamma \\ 0 \end{array}\right]+\delta C_{n^{\prime }}^{n}\left[\begin{array}{c} 0\\ -\gamma \\ 0 \end{array}\right]$$

The expression of measurement noise is obtained:

(62) $$\left[\begin{array}{c} w_{N}\\ 0\\ w_{E} \end{array}\right]=\delta C_{n^{\prime }}^{n}\left[\begin{array}{c} 0\\ -\gamma \\ 0 \end{array}\right]$$

Suppose $\delta C_{n^{\prime }}^{n}$ has the following form:

(63) $$\delta C_{n^{\prime }}^{n}=\left[\begin{array}{ccc} \delta c_{11} & \delta c_{12} & \delta c_{13}\\ \delta c_{21} & \delta c_{22} & \delta c_{23}\\ \delta c_{31} & \delta c_{32} & \delta c_{33} \end{array}\right]$$

The model of measurement noise is obtained, this is the foundation of estimating accelerometer bias in this paper:

(64) $$\begin{array}{l} w_{N}=\delta c_{12}\cdot \left(-\gamma \right)\\ w_{E}=\delta c_{32}\cdot \left(-\gamma \right) \end{array}$$

Substituting Equation (64) into Equation (43), the model of measurement error is rearranged as:

(65) $$\left[\begin{array}{c} \delta g_{N}^{n}\\ \delta g_{E}^{n} \end{array}\right]=\left[\begin{array}{c} b_{a,N}^{n}-\delta c_{12}\cdot \left(\gamma \right)\\ b_{a,E}^{n}-\delta c_{32}\cdot \left(\gamma \right) \end{array}\right]$$

### 4.2. Parameters of the Model of Measurement Noise

$\delta c_{12}$ and $\delta c_{32}$ are derived below. According Equations (50)–(57), the elements of the quaternion are also the functions of measured values of horizontal gravity disturbance. These elements will also contain some error due to the measurement noise as follows:

(66) $$\begin{array}{ccc} {\tilde{q}}_{0}=q_{0}+\delta q_{0} & {\tilde{q}}_{1}=q_{1}+\delta q_{1} & {\tilde{q}}_{3}=q_{3}+\delta q_{3} \end{array}$$

$\delta q_{0}$, $\delta q_{1}$ and $\delta q_{3}$ are the errors caused only by the measurement noise. Substituting Equation (66) into Equation (57), we can get:

(67) $$\begin{array}{cc} \delta c_{12}=-2\left(q_{0}\delta q_{3}+\delta q_{0}q_{3}\right) & \delta c_{32}=2\left(q_{0}\delta q_{1}+\delta q_{0}q_{1}\right) \end{array}$$

(68) $$\delta C_{n^{\prime }}^{n}=\left[\begin{array}{ccc} 2\left(q_{0}\cdot \delta q_{0}+q_{1}\cdot \delta q_{1}-q_{3}\cdot \delta q_{3}\right) & -2\left(q_{0}\delta q_{3}+\delta q_{0}q_{3}\right) & 2\left(q_{1}\delta q_{3}+\delta q_{1}q_{3}\right)\\ 2\left(q_{0}\delta q_{3}+\delta q_{0}q_{3}\right) & 2\left(q_{0}\cdot \delta q_{0}-q_{1}\cdot \delta q_{1}-q_{3}\cdot \delta q_{3}\right) & 2\left(q_{0}\delta q_{1}+\delta q_{0}q_{1}\right)\\ 2\left(q_{1}\delta q_{3}+\delta q_{1}q_{3}\right) & 2\left(q_{0}\delta q_{1}+\delta q_{0}q_{1}\right) & 2\left(q_{0}\cdot \delta q_{0}-q_{1}\cdot \delta q_{1}+q_{3}\cdot \delta q_{3}\right) \end{array}\right]$$

The model of measurement error is rearranged as:

(69) $$\left[\begin{array}{c} \delta g_{N}^{n}\\ \delta g_{E}^{n} \end{array}\right]=\left[\begin{array}{c} b_{a,N}^{n}+\left[2\left(q_{0}\delta q_{3}+\delta q_{0}q_{3}\right)\right]\cdot \left(\gamma \right)\\ b_{a,E}^{n}-\left[2\left(q_{0}\delta q_{1}+\delta q_{0}q_{1}\right)\right]\cdot \left(\gamma \right) \end{array}\right]$$

The next work is to derivate the expression of $\delta q_{i,i=0,1,3}$ through the following partial differential equations:

(70) $$\begin{array}{ccc} \delta q_{0}=\left(\frac{\partial q_{0}}{\partial \xi }\right)\delta \xi +\left(\frac{\partial q_{0}}{\partial \eta }\right)\delta \eta & \delta q_{1}=\left(\frac{\partial q_{1}}{\partial \xi }\right)\delta \xi +\left(\frac{\partial q_{1}}{\partial \eta }\right)\delta \eta & \delta q_{3}=\left(\frac{\partial q_{3}}{\partial \xi }\right)\delta \xi +\left(\frac{\partial q_{3}}{\partial \eta }\right)\delta \eta \end{array}$$

According to the Equations (50)–(57), these partial derivatives are obtained:

(71) $$\begin{array}{cc} f\left(\xi ,\eta \right)=1/\sqrt{1+\xi^{2}+\eta^{2}} & g\left(f\right) \end{array}=\arccos(f)$$

(72) $$\frac{\partial q_{0}}{\partial \xi }=\left(\frac{-\sin\left(g\left(f\right)/2\right)\cdot \xi }{2\left(1+\eta^{2}+\xi^{2}\right)\sqrt{\eta^{2}+\xi^{2}}}\right)$$

(73) $$\frac{\partial q_{0}}{\partial \eta }=\frac{-\sin\left(g\left(f\right)/2\right)\cdot \eta }{2\left(1+\eta^{2}+\xi^{2}\right)\sqrt{\eta^{2}+\xi^{2}}}$$

(74) $$\frac{\partial q_{1}}{\partial \xi }=\frac{\left(\xi \cdot \eta \right)}{\left(\eta^{2}+\xi^{2}\right)}\left[\frac{-\cos(g\left(f\right)/2)}{2\left(1+\eta^{2}+\xi^{2}\right)}+\frac{\sin(g\left(f\right)/2)}{\sqrt{\xi^{2}+\eta^{2}}}\right]$$

(75) $$\frac{\partial q_{1}}{\partial \eta }=-\frac{\cos\left(g\left(f\right)/2\right)\cdot \eta^{2}}{2\left(1+\xi^{2}+\eta^{2}\right)\left(\xi^{2}+\eta^{2}\right)}-\frac{\sin(g\left(f\right)/2)\cdot \left(\xi^{2}\right)}{{\left(\xi^{2}+\eta^{2}\right)}^{3/2}}$$

(76) $$\frac{\partial q_{3}}{\partial \xi }=\frac{\cos(g\left(f\right)/2)\cdot \xi^{2}}{2\left(1+\eta^{2}+\xi^{2}\right)\cdot \left(\eta^{2}+\xi^{2}\right)}+\frac{\sin(g(f))\cdot \left(\eta^{2}\right)}{{\left(\xi^{2}+\eta^{2}\right)}^{3/2}}$$

(77) $$\frac{\partial q_{3}}{\partial \eta }=\frac{\cos\left(g\left(f\right)/2\right)\xi \cdot \eta }{2\left(1+\xi^{2}+\eta^{2}\right)\left(\xi^{2}+\eta^{2}\right)}-\sin(g\left(f\right)/2)\cdot \left(\xi \cdot \eta \right){\left(\xi^{2}+\eta^{2}\right)}^{-3/2}$$

### 4.3. Estimation Model of the Accelerate Bias

Finally, based on the model of measurement noise, the model of measurement error is rearranged as follows:

(78) $$\left[\begin{array}{c} \delta g_{N}^{n}\\ \delta g_{E}^{n} \end{array}\right]=\left[\begin{array}{cccc} 2\gamma \cdot \Phi_{1} & 2\gamma \cdot \Phi_{2} & 1 & 0\\ -2\gamma \cdot \Phi_{3} & -2\gamma \cdot \Phi_{4} & 0 & 1 \end{array}\right]\left[\begin{array}{c} \delta \xi \\ \delta \eta \\ b_{a,N}^{n}\\ b_{a,E}^{n} \end{array}\right]$$

(79) $$\Phi_{1}=\left\{\cos\left(\frac{g\left(f\right)}{2}\right)\left(\frac{\partial q_{3}}{\partial \xi }\right)+\sin\left(\frac{g\left(f\right)}{2}\right)\cdot \left(\frac{\xi }{\sqrt{\xi^{2}+\eta^{2}}}\right)\left(\frac{\partial q_{0}}{\partial \xi }\right)\right\}$$

(80) $$\Phi_{2}=\left\{\cos\left(\frac{g\left(f\right)}{2}\right)\left(\frac{\partial q_{3}}{\partial \eta }\right)+\sin\left(\frac{g\left(f\right)}{2}\right)\cdot \left(\frac{\xi }{\sqrt{\xi^{2}+\eta^{2}}}\right)\left(\frac{\partial q_{0}}{\partial \eta }\right)\right\}$$

(81) $$\Phi_{3}=\left\{\cos\left(\frac{g\left(f\right)}{2}\right)\left(\frac{\partial q_{1}}{\partial \xi }\right)+\sin\left(\frac{g\left(f\right)}{2}\right)\cdot \left(\frac{-\eta }{\sqrt{\xi^{2}+\eta^{2}}}\right)\cdot \left(\frac{\partial q_{0}}{\partial \xi }\right)\right\}$$

(82) $$\Phi_{4}=\left\{\cos\left(\frac{g\left(f\right)}{2}\right)\left(\frac{\partial q_{1}}{\partial \eta }\right)+\sin\left(\frac{g\left(f\right)}{2}\right)\cdot \left(\frac{-\eta }{\sqrt{\xi^{2}+\eta^{2}}}\right)\cdot \left(\frac{\partial q_{0}}{\partial \eta }\right)\right\}$$

δξ and δη can be regarded as the measurement errors of DOV only due to the measurement noise $w_{N}$ and $w_{E}$, and the contribution of δξ and δη to the measurement error of horizontal gravity disturbance is associated with the variation of gravity field, these connections are represented by $\Phi_{i,i=1,2,3,4}$.

## 5. Simulation Results

### 5.1. Estimation Methods of Accelerometer Bias

The model of measurement error, as Equation (78), can be utilized in the method of least squares to estimate the accelerometer bias, the elements $\Phi_{i,i=1,2,3,4}$ change with the variation of gravity field, then uncorrelated observation equations are built to ensure the numerical stability of solving the pseudo-inverse in the least squares. Assuming that *N* times measurements of horizontal gravity disturbance are made, then there are 2*N* observation equations built as below:

(83) $$z_{2N\times 1}=H_{2N\times 4}\cdot x$$

(84) $$x={\left[\begin{array}{cccc} x_{1} & x_{2} & x_{3} & x_{4} \end{array}\right]}^{T}={\left[\begin{array}{cccc} \delta \xi & \delta \eta & b_{a,N}^{n} & b_{a,E}^{n} \end{array}\right]}^{T}$$

(85) $$z_{2N\times 1}=\left[\begin{array}{ccccc} \delta g_{N}^{n}\left(1\right) & \delta g_{E}^{n}\left(1\right) & \cdots & \delta g_{N}^{n}\left(N\right) & \delta g_{E}^{n}\left(N\right) \end{array}\right]$$

(86) $$H_{2N\times 4}=\left[\begin{array}{cccc} 2\gamma \left(1\right)\cdot \Phi_{1}\left(1\right) & 2\gamma \left(1\right)\cdot \Phi_{2}\left(1\right) & 1 & 0\\ -2\gamma \left(1\right)\cdot \Phi_{3}\left(1\right) & -2\gamma \left(1\right)\cdot \Phi_{4}\left(1\right) & 0 & 1\\ \vdots & \vdots & \vdots & \vdots \\ 2\gamma \left(N\right)\cdot \Phi_{1}\left(N\right) & 2\gamma \left(N\right)\cdot \Phi_{2}\left(N\right) & 1 & 0\\ -2\gamma \left(N\right)\cdot \Phi_{3}\left(N\right) & -2\gamma \left(N\right)\cdot \Phi_{4}\left(N\right) & 0 & 1 \end{array}\right]$$

where $z_{2N\times 1}$ is the measurement error of horizontal gravity disturbance which is calculated by subtracting the true values from the measurement values, and the subscript means the vector has 2N rows and one column. $H_{2N\times 4}$ is the observation matrix which has 2*N* rows and four columns, $\Phi_{i,i=1,2,3,4}$ can be determined by substituting true values of DOV into Equations (79)–(82). γ(i) is the norm of the normal gravity vector.

The accelerometer bias can be estimated by least squares as follows. ${\hat{b}}_{a,N}^{n}$ and ${\hat{b}}_{a,E}^{n}$ are the estimations of the accelerometer biases:

(87) $$x={\left(H_{2N\times 4}^{T}\cdot H_{2N\times 4}\right)}^{-1}\cdot H_{2N\times 4}^{T}\cdot z_{2N\times 1}$$

(88) $$\begin{array}{cc} {\hat{b}}_{a,N}^{n}=x_{3} & {\hat{b}}_{a,E}^{n}=x_{4} \end{array}$$

### 5.2. Simulation of Horizontal Gravity Disturbance

**Figure 6:**
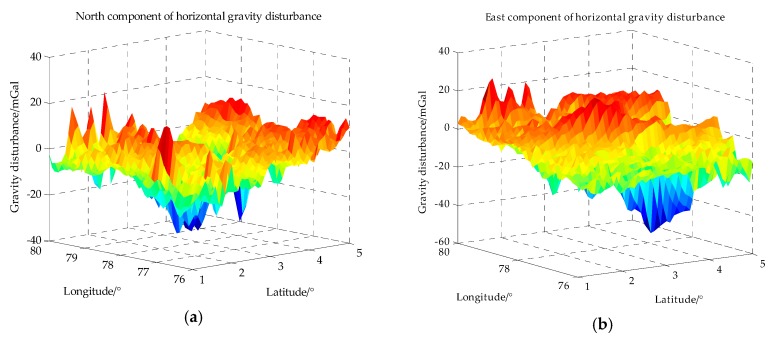
The horizontal gravity disturbance in the simulation area. (**a**) is the north component of the horizontal gravity disturbance and (**b**) is the east component of the horizontal gravity disturbance. (1 mGal = 10^−5^ m/s^2^).

From the model of measurement error, it can be known that the contribution of measurement noise to measurement error changes with the variation of gravity field. In this simulation, an area with moderate variation of gravity field is chosen whose latitude range is from 1° N to 5° N and longitude range is from 76° E to 80° E, as shown in Table 1. The horizontal gravity disturbance in this area is calculated based on SHM as described in Section 2, the SHM adopted here is EGM2008 whose the maximum degree is 2190. The horizontal gravity disturbance in the simulation area is calculated with maximum degree and the grid spacing is 5 n mile, the horizontal gravity disturbance in this simulation area is shown in Figure 6.

**Figure 7:**
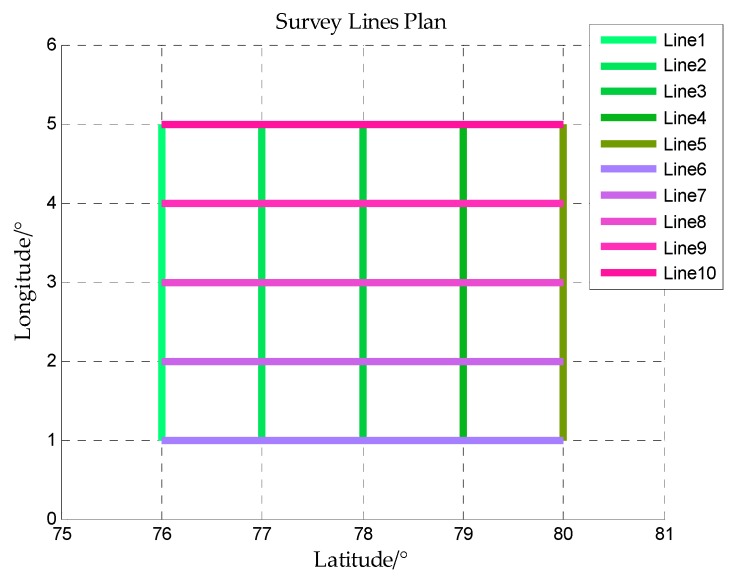
The survey lines in the simulation area.

**Figure 8:**
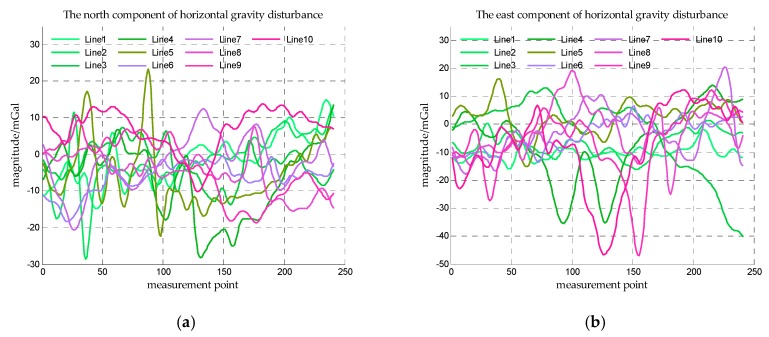
The horizontal gravity disturbance on the survey lines. (**a**) is the north component of horizontal gravity disturbance on survey lines and (**b**) is the east component of horizontal gravity disturbance on survey lines.

The ship is assumed to sail along some straight lines in this area, these straight lines are called survey lines. The gravity disturbance measurement is implemented along these survey lines. Five latitude lines and five longitude lines in this area are taken as survey lines as shown in Figure 7 and the horizontal gravity disturbance on the survey lines is shown in Figure 8.

### 5.3. Simulation Results

In this subsection, the efficiency of the proposed estimation method will be verified by the simulations. The true values of horizontal gravity disturbances are the calculation results of SHM, and the measured values are constructed as Equation (42), then the measurement error can be obtained by subtracting the true values from the measurement values. 

The bias of accelerometer $b_{a}$ is sometimes split into static component and dynamic component [34]. The static component denoted by $b_{as}$ is also known as the bias repeatability and comprises the run-to-run variation of instrument bias plus the residual fixed bias remaining after calibration. $b_{as}$ is constant throughout an IMU operating period but varies from run to run. The dynamic component denoted by ***b****_ad_* is also known as the bias instability and varies over time and temperature:

(89) $$b_{a}=b_{as}+b_{ad}$$

The value of bias repeatability is chosen with reference to the datasheet of QA3000, which is the highest navigation-grade accelerometer from Honeywell^®^. The one-year composite repeatability of QA3000 is less than 40 mGal, this value can be regarded as the upper bound of accelerometer bias. According to the analysis in the end of Section 3, when the accelerometer bias is at the same order with horizontal gravity disturbance, estimation of accelerometer bias is a crucial issue in the horizontal gravity disturbance compensation, so the magnitude of accelerometer bias is carefully chosen based on the magnitude of horizontal gravity disturbance in the simulation area. The statistical values of the horizontal gravity disturbance on the survey lines are list in Table 2.

**Figure 9:**
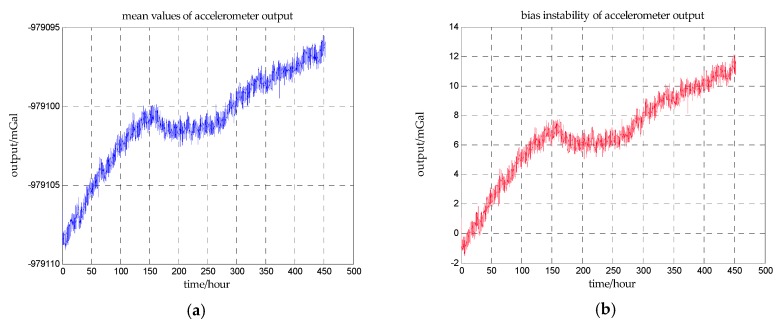
(**a**) is the mean values of the accelerometer outputs every one hundred seconds, and (**b**) is the bias instability of the accelerometer.

The values of bias instability are chosen with reference to the measured data of a high-quality accelerometer. A long term static experiment for investigating the bias instability characteristics of this accelerometer is carried out in December 2016. The output of the accelerometer is recorded for 450 h and the accelerometer is mounted on a stable platform. The sampling frequency is 200 Hz and the mean values of outputs are calculated every one hundred seconds as shown in Figure 9a. The drift of the accelerometer output can be obtained by subtracting the first mean value form the subsequent mean values as shown in Figure 9b.

It can be seen from Figure 9 that the characteristics of bias instability changes over time. From the beginning to the 150th hour, the bias instability of accelerometer is a linear drift, and the drift rate is approximately 1.31 mGal/day. From the 150th hour to the 250th hour, the bias instability changes to a second-order curve. From the 250th hour to the end, the bias instability changes to a linear drift again, and the drift rate is approximately 0.59 mGal/day. 

**Figure 10:**
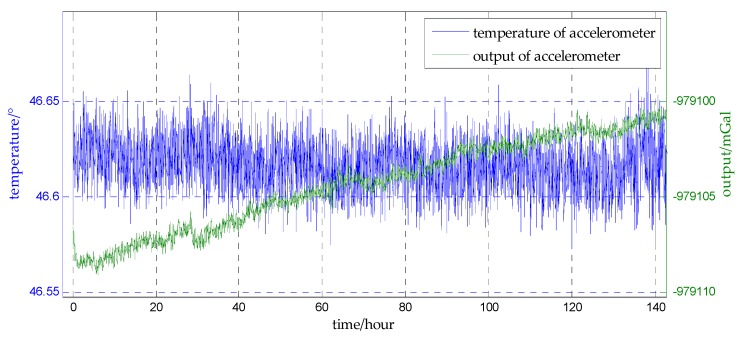
Comparing the temperature of the accelerometer with the output of the accelerometer.

**Figure 11:**
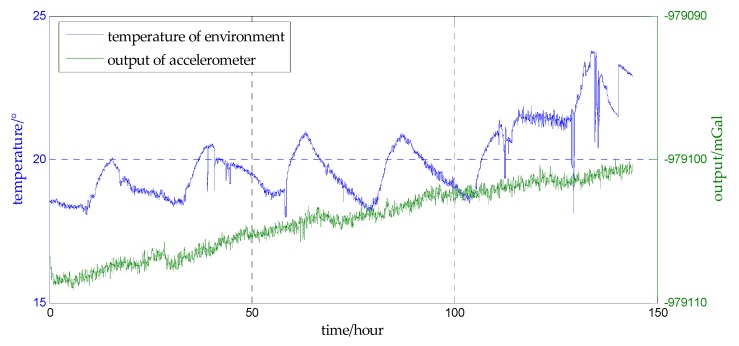
Comparing the temperature of the environment with the output of the accelerometer.

Maintaining a nearly constant and stable temperature of the IMU improves its accuracy during calibration and operation, as temperature stability is directly related to the sensor accuracy. A multi-point thermal control is used in this experiment to improve the precision of the accelerometer, and the temperatures of the accelerometer and the environment are recorded for the first 140 h. The connections between the outputs of the accelerometer and the measured temperatures are shown in Figure 10 and Figure 11. Comparing Figure 10 and Figure 11, there is no cyclic change in the temperature of the accelerometer while the temperature of the environment changing day and night, which means under the control the temperature of the accelerometer is stable. And there is no obvious correlation between the output of the accelerometer and the temperature of the accelerometer. This indicates that the bias instability of accelerometer is approximately irrelevant to the temperature in this experiment, and can be considered to be a linear drift over time, then the model of bias instability can be built as Equation (90), where t is time, $k_{ad,N}^{n}$ is the drift rate of north component and $k_{ad,E}^{n}$ is the drift rate of the east component:

(90) $$\begin{array}{l} b_{ad,N}^{n}=k_{ad,N}^{n}\cdot t\\ b_{ad,E}^{n}=k_{ad,E}^{n}\cdot t \end{array}$$

The bias repeatability is supposed to be equal to the mean value or median of all the survey lines, and the bias instability is supposed to be equal to the first drift rate in the above static experiment, 1.31 mGal/day. The measurement noise ranges from 1 mGal to 20 mGal, which is a reasonable range for high precision inertial sensors and GNSS receiver. The signal-to-noise ratio (SNR) is defined as:

(91) $$SNR=\begin{array}{cc} 20\log_{10}\left(\frac{magnitude\;of\;accelerometer\;bias}{magnitude\;of\;measurement\;noise}\right) & dB \end{array}$$

Three simulations are designed here to verify the validity of the proposed method as described in Table 3, and the differences among these simulations are the types and scales of the accelerometer bias.

**Figure 12:**
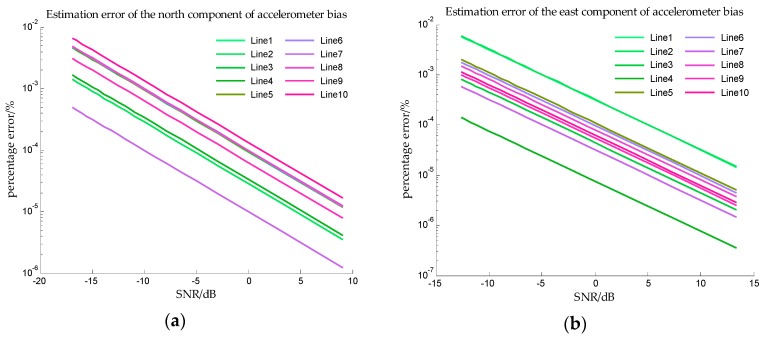
The estimation error of accelerometer bias in simulation I. (**a**) is the estimation error of the north component of the accelerometer bias, and (**b**) is the estimation error of the east component of the accelerometer bias.

**Figure 13:**
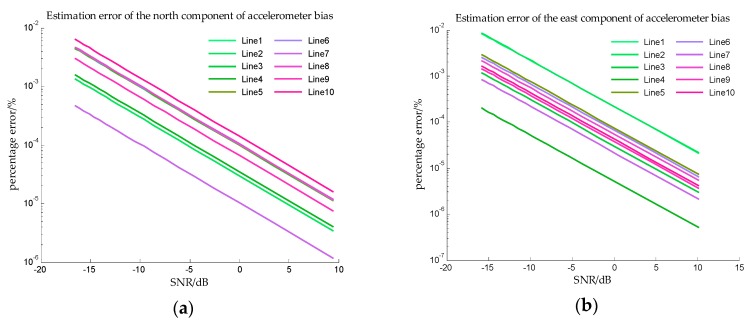
The estimation error of accelerometer bias in simulation II. (**a**) is the estimation error of the north component of the accelerometer bias, and (**b**) is the estimation error of the east component of the accelerometer bias.

The estimation errors of accelerometer bias in Simulation I are shown in Figure 12 and the estimation errors of accelerometer bias in Simulation II are shown in Figure 13. From Figure 12 and Figure 13, the precise estimations of accelerometer bias are obtained in the proposed method. Even if the SNR is −15 dB, the estimation error is less than $10^{-2}$%. For estimating the north component of accelerometer bias, the estimation error of survey line 10 is significantly greater than the errors of other survey lines, this may be explained from Table 2, the mean value and median of survey line 10 are both large values which indicates the variation of north component of horizontal gravity disturbance on line 10 is the most drastic. The more drastic variation of gravity field, the more contribution of measurement noise to measurement error, so the weight of accelerometer bias in measurement noise is less. The decrease of the effective information in the observed quantity leads to the decrease of the estimation accuracy. This interpretation is also suitable for survey line 1, on which the variation of east component of horizontal gravity disturbance is the most drastic, and the estimation error of east component of accelerometer bias is significantly greater.

In Simulation III, some modifications are needed on Equations (83)–(86) to estimate the bias repeatability and drift rate simultaneously:

(92) $$z_{2N\times 1}=H_{2N\times 6}\cdot x$$

(93) $$x={\left[\begin{array}{cccccc} \delta \xi & \delta \eta & b_{as,N}^{n} & b_{as,E}^{n} & k_{ad,N}^{n} & k_{ad,E}^{n} \end{array}\right]}^{T}$$

(94) $$H_{2N\times 6}=\left[\begin{array}{cccccc} 2\gamma \left(1\right)\cdot \Phi_{1}\left(1\right) & 2\gamma \left(1\right)\cdot \Phi_{2}\left(1\right) & 1 & 0 & t & 0\\ -2\gamma \left(1\right)\cdot \Phi_{3}\left(1\right) & -2\gamma \left(1\right)\cdot \Phi_{4}\left(1\right) & 0 & 1 & 0 & t\\ \vdots & \vdots & \vdots & \vdots & \vdots & \vdots \\ 2\gamma \left(N\right)\cdot \Phi_{1}\left(N\right) & 2\gamma \left(N\right)\cdot \Phi_{2}\left(N\right) & 1 & 0 & t & 0\\ -2\gamma \left(N\right)\cdot \Phi_{3}\left(N\right) & -2\gamma \left(N\right)\cdot \Phi_{4}\left(N\right) & 0 & 1 & 0 & t \end{array}\right]$$

(95) $$x={\left(H_{2N\times 6}^{T}\cdot H_{2N\times 6}\right)}^{-1}\cdot H_{2N\times 6}^{T}\cdot z_{2N\times 1}$$

(96) $$\begin{array}{cccc} {\hat{b}}_{as,N}^{n}=x_{3} & {\hat{b}}_{as,E}^{n}=x_{4} & {\hat{k}}_{ad,N}^{n}=x_{5} & {\hat{k}}_{ad,E}^{n}=x_{6} \end{array}$$

where *t* is time, ${\hat{b}}_{as,N}^{n}$ and ${\hat{b}}_{as,E}^{n}$ are the estimations of bias repeatability, ${\hat{k}}_{ad,N}$ and ${\hat{k}}_{ad,E}$ are the estimations of drift rates.

**Figure 14:**
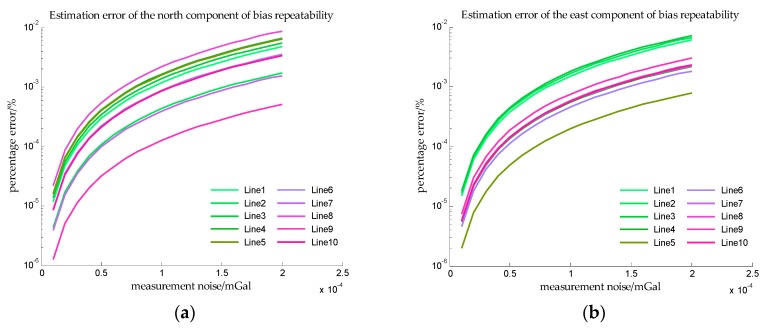
The estimation error of accelerometer bias in simulation III. (**a**) is the estimation error of the north component of the bias repeatability, and (**b**) is the estimation error of the east component of the bias repeatability.

**Figure 15:**
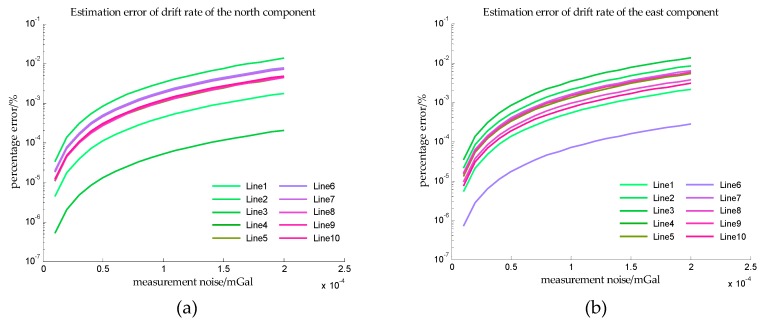
The estimation error of drift rate in simulation III. (**a**) is the estimation error of the drift rate of the north component, and (**b**) is the estimation error of the drift rate of the east component.

In order to obtain more observations to improve the accuracy of estimation, the speed of the ship is assumed to be 5 knot, and it can be seen from Table 2 that the sailing time is 48 h. The estimation error in Simulation III are shown in Figure 14 and Figure 15.

In Simulation III, the accelerometer bias is time varying, so the *x*-axis is measurement noise instead of SNR. And from Figure 14 and Figure 15, the estimation errors of bias repeatability are less than 10^−2^%, and the estimation errors of drift rate are also less than 10^−2^%. The results of simulation III indicate that the proposed method can accurately estimate the bias repeatability and bias instability simultaneously.

It should be noted that, in the above simulations, although a precise estimation of accelerometer bias is obtained, the length of the survey line is 240 n miles, which is too long and not suitable for practical applications. Further simulations are carried out for the typical application scenario. 

**Figure 16:**
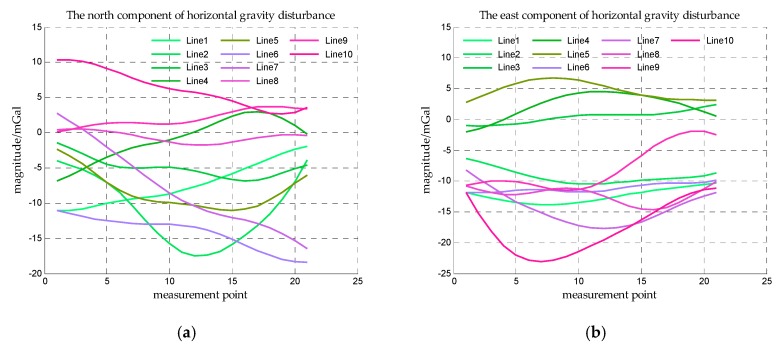
The horizontal gravity disturbance on the short survey lines. (**a**) is the north component of horizontal gravity disturbance on the short survey lines and (**b**) is the east component of horizontal gravity disturbance on the short survey lines.

The ship’s speed is generally 20 knots, and the time for estimating accelerometer bias is assumed to be one hour. The sample period of strapdown gravimeter is generally 160~300 s [25], then the distance between the measurement points is supposed to be 1 n mile. Based on the three assumed conditions, the length of survey line is supposed to be 20 n miles and the coordinate range of these short survey lines are list in Table 4 and the horizontal gravity disturbances on the short survey lines are shown in Figure 16. 

Three simulations are designed here to verify the validity of the proposed method on the short survey lines as described in Table 5, and the differences among these simulations are also the types and scales of the accelerometer bias. The measurement noise is consistent with the previous setting.

**Figure 17:**
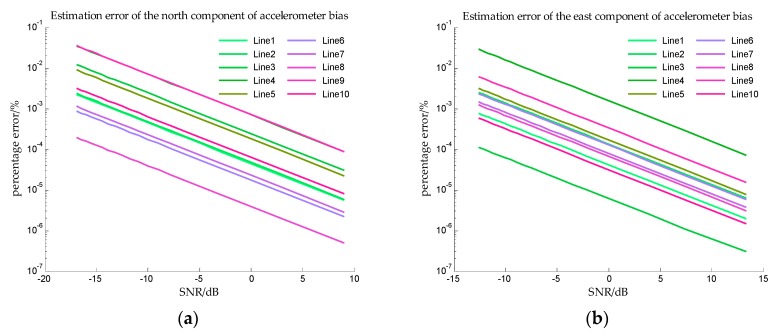
The estimation error of accelerometer bias on the short survey lines in simulation IV. (**a**) Is the estimation error of the north component of the accelerometer bias, and (**b**) is the estimation error of the east component of the accelerometer bias.

**Figure 18:**
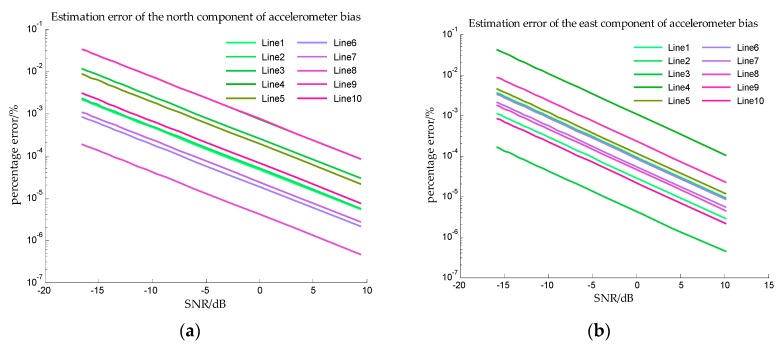
The estimation error of accelerometer bias on the short survey lines in simulation V. (**a**) Is the estimation error of the north component of the accelerometer bias, and (**b**) is the estimation error of the east component of the accelerometer bias.

The estimation errors of accelerometer bias on the short survey lines in Simulation IV are shown in Figure 17 and the estimation errors of accelerometer bias on the short survey lines in Simulation V are shown in Figure 18. From Figure 17 and Figure 18, it can be seen that the maximum estimation error of accelerometer bias is less than $10^{-1}$% in the typical application scenario. It can be known from Table 4 that there are only twenty-one measured values of the horizontal gravity disturbance in this application scenario. Compared the estimation errors with those of the long survey lines, the reduction of estimation accuracy is due to the reduction of observation.

**Figure 19:**
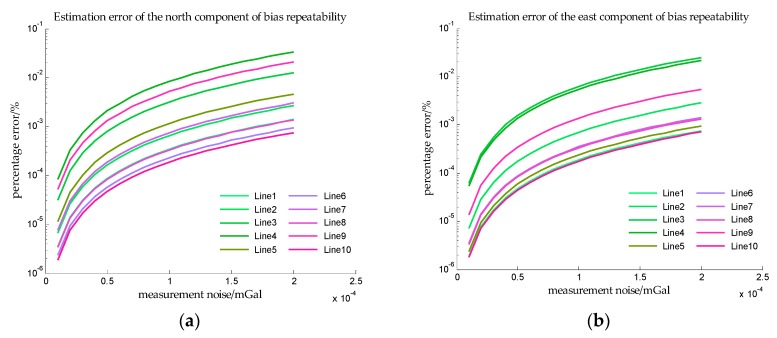
The estimation error of the bias repeatability on the short survey lines in simulation VI. (**a**) Is the estimation error of the north component of the bias repeatability, and (**b**) is the estimation error of the east component of the bias repeatability.

**Figure 20:**
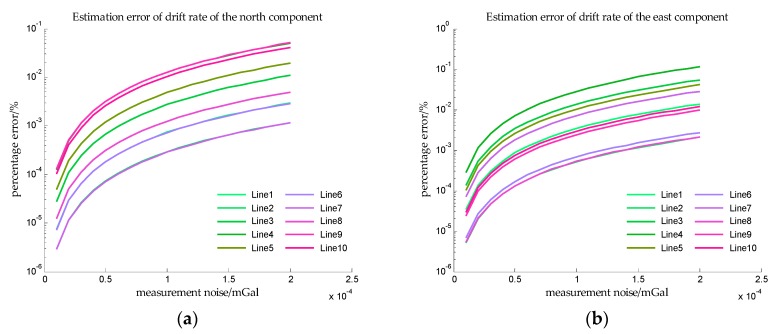
The estimation error of drift rate on the short survey lines in simulation VI. (**a**) is the estimation error of the drift rate of the north component, and (**b**) is the estimation error of the drift rate of the east component.

The estimation errors of bias repeatability in Simulation VI are shown in Figure 19 and the estimation errors of bias instability in Simulation VI are shown in Figure 20. It can be found that the estimation errors of bias repeatability are less than 10^−1^%, and the estimation errors of drift rate are also less than 10^−1^%. The results in Simulation VI indicate that the proposed method can accurately estimate the bias repeatability and bias instability simultaneously in the typical application scenario. Also due to the reduction of the observations, the estimation errors of bias repeatability and bias instability in simulation VI are both larger than those in Simulation III.

It should be noted that in the above simulations the bias of the gyroscope is assumed to be zero. In practice, gyroscope bias may affect the estimation accuracy of the proposed method, as gyroscope bias will produce some specific force measurement error. In other words, the estimation may be a combination of accelerometer bias and gyroscope bias. As described in [35], observability is crucial for estimation of gyroscope bias in INS/GNSS. From the observability analysis, biases of the horizontal gyroscopes can be accurately estimated. But it’s difficult to accurately estimate the bias of the vertical gyroscope due to the poor observability. Thus, the estimation accuracy of the proposed approach may be affected by the bias of the vertical gyroscope.

## 6. Conclusions

Horizontal gravity disturbance produces a restriction on the achievable accuracy of INS. Although based on SHM horizontal gravity disturbances can be accurately obtained and compensated, the effect of compensation will decrease due to the accelerometer bias. Through the analysis, it is found that estimating the accelerometer bias is a necessary condition to ensure the effect of horizontal gravity disturbance compensation, especially when the accelerometer bias is of the same order as the horizontal gravity disturbance. The method of estimating the accelerometer bias from the gravity vector measurement is proposed, and the model of measurement noise is derived to separate the accelerometer bias from the measurement error. Simulation results verify the effect of the proposed method, and a precise estimation of accelerometer bias is obtained in the typical application scenario.

## Figures and Tables

**Table 1:** The coordinate range of the survey lines.

Line No.	Latitude Range	Longitude Range	Grid Spacing
Line 1	1° N~5° N	76° E	5 n mile
Line 2	1° N~5° N	77° E	5 n mile
Line 3	1° N~5° N	78° E	5 n mile
Line 4	1° N~5°	79° E	5 n mile
Line 5	1° N~5° N	80° E	5 n mile
Line 6	1° N	76° E~80° E	5 n mile
Line 7	2° N	76° E~80° E	5 n mile
Line 8	3° N	76° E~80° E	5 n mile
Line 9	4° N	76° E~80° E	5 n mile
Line 10	5° N	76° E~80° E	5 n mile

**Table 2:** The statistical values of horizontal gravity disturbance on the survey lines.

Line No.	Mean Value/mGal		Median/mGal	
	North Component	East Component	North Component	East Component
1	−1.21	−10.36	−1.55	−10.57
2	−1.61	−6.87	−2.42	−6.64
3	−2.61	−3.20	−2.97	1.25
4	−7.07	−6.27	−3.43	−3.23
5	−5.50	2.58	−8.65	3.32
6	−6.42	−3.62	−5.70	−2.17
7	−2.24	−2.62	−2.01	−3.21
8	−4.96	−2.58	−4.44	−3.10
9	−3.61	−5.79	−2.92	−3.50
10	6.84	−7.92	8.35	−6.84
	Mean value of all survey lines		Median of all survey lines	
	−2.84	−4.67	−2.97	−3.23

**Table 3:** Simulations on the survey lines.

Simulation No.	Description
Simulation I	Only bias repeatability is considered and is equal to the mean value of all the survey lines
Simulation II	Only bias repeatability is considered and is equal to the median value of all the survey lines
Simulation III	Bias repeatability and bias instability are both considered, where bias repeatability is equal to the mean value of all the survey lines and drift rate is 1.31 mGal/day

**Table 4:** The coordinate range of the short survey lines.

Line No.	Latitude Range	Longitude Range	Spacing
Line 1	1° N~1°20′ N	76° E	1 n mile
Line 2	1° N~1°20′ N	77° E	1 n mile
Line 3	1° N~1°20′ N	78° E	1 n mile
Line 4	1° N~1°20′ N	79° E	1 n mile
Line 5	1° N~1°20′ N	80° E	1 n mile
Line 6	1° N	76° E~76°20′ E	1 n mile
Line 7	2° N	76° E~76°20′ E	1 n mile
Line 8	3° N	76° E~76°20′ E	1 n mile
Line 9	4° N	76° E~76°20′ E	1 n mile
Line 10	5° N	76° E~76°20′ E	1 n mile

**Table 5:** Simulations on the short survey lines.

Simulation No.	Description
Simulation IV	On the short survey lines, only bias repeatability is considered and is equal to the mean value of all the survey lines
Simulation V	On the short survey lines, only bias repeatability is considered and is equal to the median value of all the survey lines
Simulation VI	On the short survey lines, bias repeatability and bias instability are both considered, where bias repeatability is equal to the mean value of all the survey lines and drift rate is 1.31 mGal/day
